# VICTORIA: VIrtual neck Curve and True Ostium Reconstruction of Intracranial Aneurysms

**DOI:** 10.1007/s13239-021-00535-w

**Published:** 2021-06-07

**Authors:** Philipp Berg, Benjamin Behrendt, Samuel Voß, Oliver Beuing, Belal Neyazi, Ibrahim Erol Sandalcioglu, Bernhard Preim, Sylvia Saalfeld

**Affiliations:** 1 Department of Fluid Dynamics and Technical Flows, University of Magdeburg, Magdeburg, Germany; 2 Department of Radiology, AMEOS Hospital, Bernburg, Germany; 3 Department of Neurosurgery, University Hospital of Magdeburg, Magdeburg, Germany; 4 Department of Simulation and Graphics, University of Magdeburg, Magdeburg, Germany

**Keywords:** Intracranial aneurysm, Neck curve, Rupture risk assessment, Hemodynamics, VICTORIA

## Abstract

**Purpose:**

For the status evaluation of intracranial aneurysms (IAs), morphological and hemodynamic parameters can provide valuable information. For their extraction, a separation of the aneurysm sac from its parent vessel is required that yields the neck curve and the ostium. However, manual and subjective neck curve and ostium definitions might lead to inaccurate IA assessments.

**Methods:**

The research project VICTORIA was initiated, allowing users to interactively define the neck curve of five segmented IA models using a web application. The submitted results were qualitatively and quantitatively compared to identify the minimum, median and maximum aneurysm surface area. Finally, image-based blood flow simulations were carried out to assess the effect of variable neck curve definitions on relevant flow- and shear-related parameters.

**Results:**

In total, 55 participants (20 physicians) from 18 countries participated in VICTORIA. For relatively simple aneurysms, a good agreement with respect to the neck curve definition was found. However, differences among the participants increased with increasing complexity of the aneurysm. Furthermore, it was observed that the majority of participants excluded any small arteries occurring in the vicinity of an aneurysm. This can lead to non-negligible deviations among the flow- and shear-related parameters, which need to be carefully evaluated, if quantitative analysis is desired. Finally, no differences between participants with medical and non-medical background could be observed.

**Conclusions:**

VICTORIAs findings reveal the complexity of aneurysm neck curve definition, especially for bifurcation aneurysms. Standardization appears to be mandatory for future sac-vessel-separations. For hemodynamic simulations a careful neck curve definition is crucial to avoid inaccuracies during the quantitative flow analysis.

**Supplementary Information:**

The online version contains supplementary material available at 10.1007/s13239-021-00535-w.

## Introduction

Intracranial aneurysms (IAs) are complexly shaped malformations of the cerebral vasculature, which are exposed to the risk of a rupture with a subsequent subarachnoid hemorrhage.^6^,^7^ For the assessment of the individual IA status, simple measurements of the size, easy clinical scores such as the PHASES score or more advanced morphological parameters are typically used.^16^,^24^,^25^ Furthermore, therapy planning (e.g., using minimally-invasive flow diverter stents) requires a detailed knowledge of the individual IA neck size to select an appropriate treatment strategy and device, respectively.^15^,^26^ To account for both clinical diagnosis and treatment planning, the patient-specific aneurysm can be segmented from high-resolution image data and the sac is separated from the corresponding parent vessel. This requires a sufficient 3D model, which is clearly more suitable compared to 2D projected images that can suffer from potential interobserver variability and image-dependency (i.e. the viewing angle of the 2D projections).^29^,^35^

During the last decade, an increasing number of investigations focusing on the assessment of morphological and hemodynamic parameters with partly diverging conclusions was published.^9^,^21^,^23^,^32^,^37^ Although this is basically a result of the different evaluation procedures^38^ rather than deficiencies in the underlying numerical methods, this led to partly reservations and limited trust among physicians.

One reason for the large differences in the parameter assessment was that the separation between the parent vessel and the aneurysm sac (i.e. the ostium) strongly varied.^3^ Separating the IA from the healthy parent vessel was often realized by simply using a (planar) cut-plane,^20^,^36^ which might be error-prone for complex IA shapes. Specifically, aneurysms with a broad and circumferential neck require reliable assessment and appropriate treatment planning. For overcoming such problems, tailored image-processing software, e.g., the vascular modeling toolkit (VMTK) was applied to obtain an individual aneurysm sac.^1^ Furthermore, we developed a semi-automatic extraction of an anatomical, bent neck curve decreasing the user-dependency and analysis inaccuracy.^30^ Having the technical tools at hand, we initiated the multidisciplinary project VICTORIA (VIrtual neck Curve and True Ostium Reconstruction of Intracranial Aneurysms) aiming towards a standardization of IA neck curve and ostium reconstruction. Based on this interactive web application, we were able to gather expert-knowledge from clinicians as well as non-clinicians from all around the world enabling a multidisciplinary overview on the evaluation of IAs differing in size, shape and location. Hence, the corresponding findings will be beneficial for decreasing the chances of insufficient analyses and in consequence unreliable conclusions with respect to IA status assessment and therapy planning.

## Methods

### Intracranial Aneurysm Selection and Imaging

Within the VICTORIA study, participants were requested to identify the neck curve of patient-specific IA models extracted from 3D digital subtraction angiography data. To enable this task and obtain a compromise between variability and feasibility, five cases with different complexity were selected. The aneurysms were located at the middle cerebral artery (Case 1 and 5), posterior inferior cerebellar artery (Case 2 and 4) and posterior communicating artery (Case 3), respectively. The 3D imaging was performed on an Artis Q angiography system (Siemens Healthcare GmbH, Forchheim, Germany) and appropriately reconstructed and segmented afterwards.^4^,^14^

**Figure 1:**
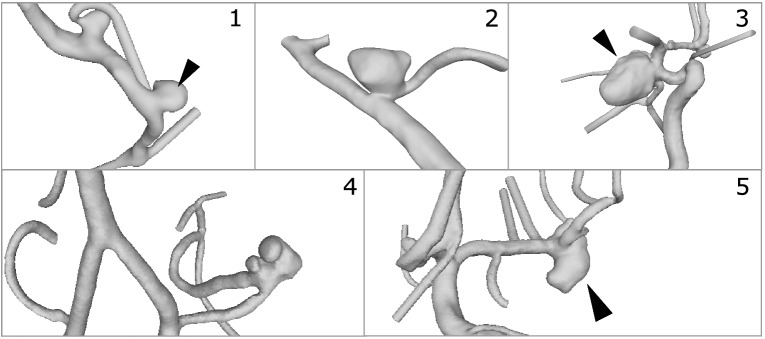
Depiction of the five patient-specific IAs used in the VICTORIA study. The aneurysms were located at the middle cerebral artery (Case 1 and 5), posterior inferior cerebellar artery (Case 2 and 4) and posterior communicating artery (Case 3), respectively. In the presence of multiple IAs, the selected one is highlighted (see arrow heads).

In Fig. 1, the final 3D IA models are presented. Further details regarding the individual morphology can be found in Table 1. Hence, we put the focus on morphological parameters that took the neck size into account.^12^,^28^

**Table 1:** Morphological information about the IAs included in the VICTORIA study, where $D_{\text{max}}$ denotes the maximum aneurysm diameter, $N_{\text{avg}}$ the average neck curve diameter, $N_{\text{max}}$ the maximum diameter and *AR* the aspect ratio.

Case	Type	$D_{\text{max}} \,({\text{mm}})$	$N_{\text{avg}}\,({\text{mm}})$	$N_{\text{max}}\,({\text{mm}})$	AR
1	Lateral	$3.76$	$2.22$	$2.48$	1.36
2	Bifurcation	$5.15$	$2.68$	$3.39$	1.42
3	Bifurcation	$15.77$	$8.94$	$10.13$	1.54
4	Lateral	$7.73$	$3.34$	$3.95$	1.69
5	Bifurcation	$13.41$	$5.44$	$6.49$	1.06

### Web Application

The VICTORIA study was conducted using a specialized web application (https://VICTORIA.cs.ovgu.de/), which consisted of two main parts: 1) a client part and 2) a server part. Between the client and the server, the data was exchanged in the JSON format and the server stored submitted data in a relational database, thus allowing for an easy sorting and filtering of the files. The rendering of the segmented surface meshes was performed using the WebGL2 API, which is available in most current desktop browsers. Furthermore, the mesh was illuminated using the Phong lighting model to increase shape perception. To ensure that the neck region was always visible and centered in the image, the user had limited control over the camera (restricted rotation, zoom and panning).

In order to recruit as many participants as possible, we provided flyers and contacted our cooperation partners via mail. For participation, only the link and a web browser was required. We also asked our peers for sharing the information within their respective networks.

#### Neck Curve Definition

**Figure 2:**
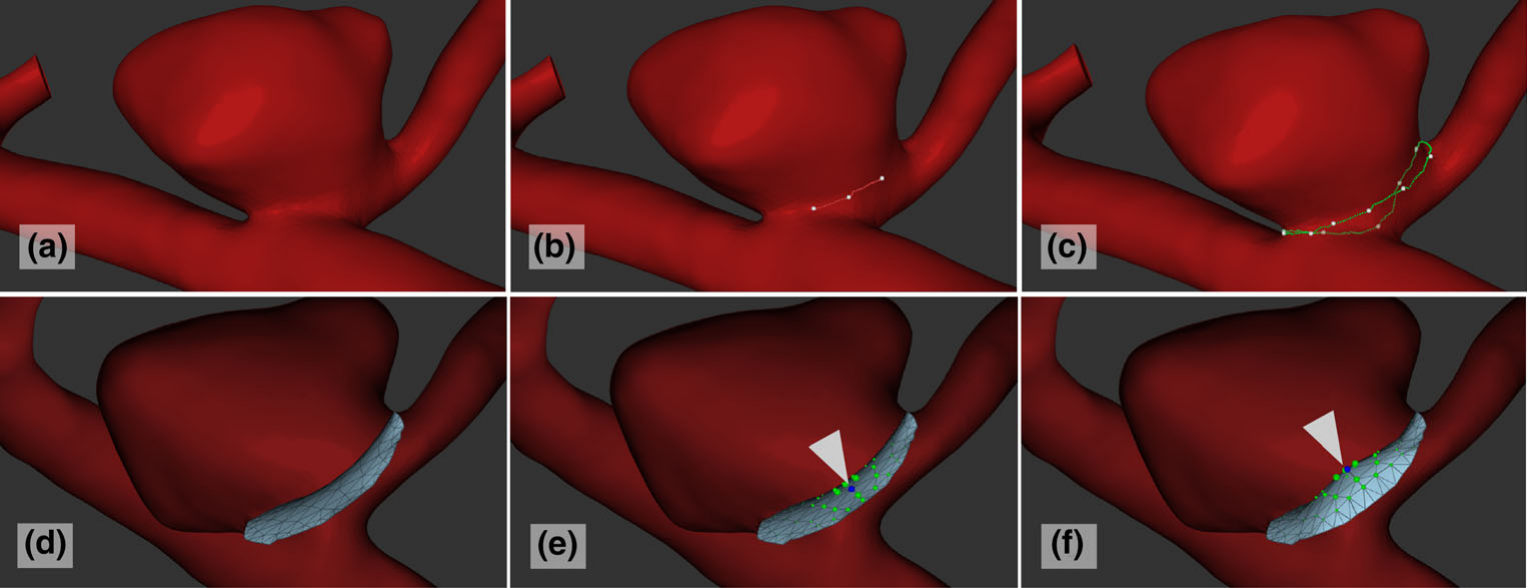
Illustration of the different steps involved in the neck curve and ostium definition: (a) 3D visualization of the surface model is shown; (b) The user can interactively select points on the aneurysm surface; (c) If the points are close to each other, the neck curve is automatically closed; (d) In the second step, an automatic ostium triangulation is provided; (e) The user can hover over the points and the active point is highlighted in blue (see arrowhead); (f) The point can be moved (including a reduced movement of its neighbors) until the user is satisfied with the ostium shape.

The first task for each participant was to draw an aneurysm neck curve onto the surface mesh by selecting arbitrary points, see Fig. 2. To connect the vertices closest to these points into a circular path, the surface triangle mesh was interpreted as a bidirectional graph. The shortest paths between the selected points were calculated using the A* algorithm by Hart *et al.*^18^

After processing all points provided by the user, the resulting list contained the shortest path connecting all neck points. In case the resulting neck curve did not match the users’ expectation, additional points could be added. Everything is implemented in JavaScript and performed entirely on the client side. Nevertheless, the complete processing runs interactively without any noticeable delay, even on less powerful devices.

**Figure 3:**
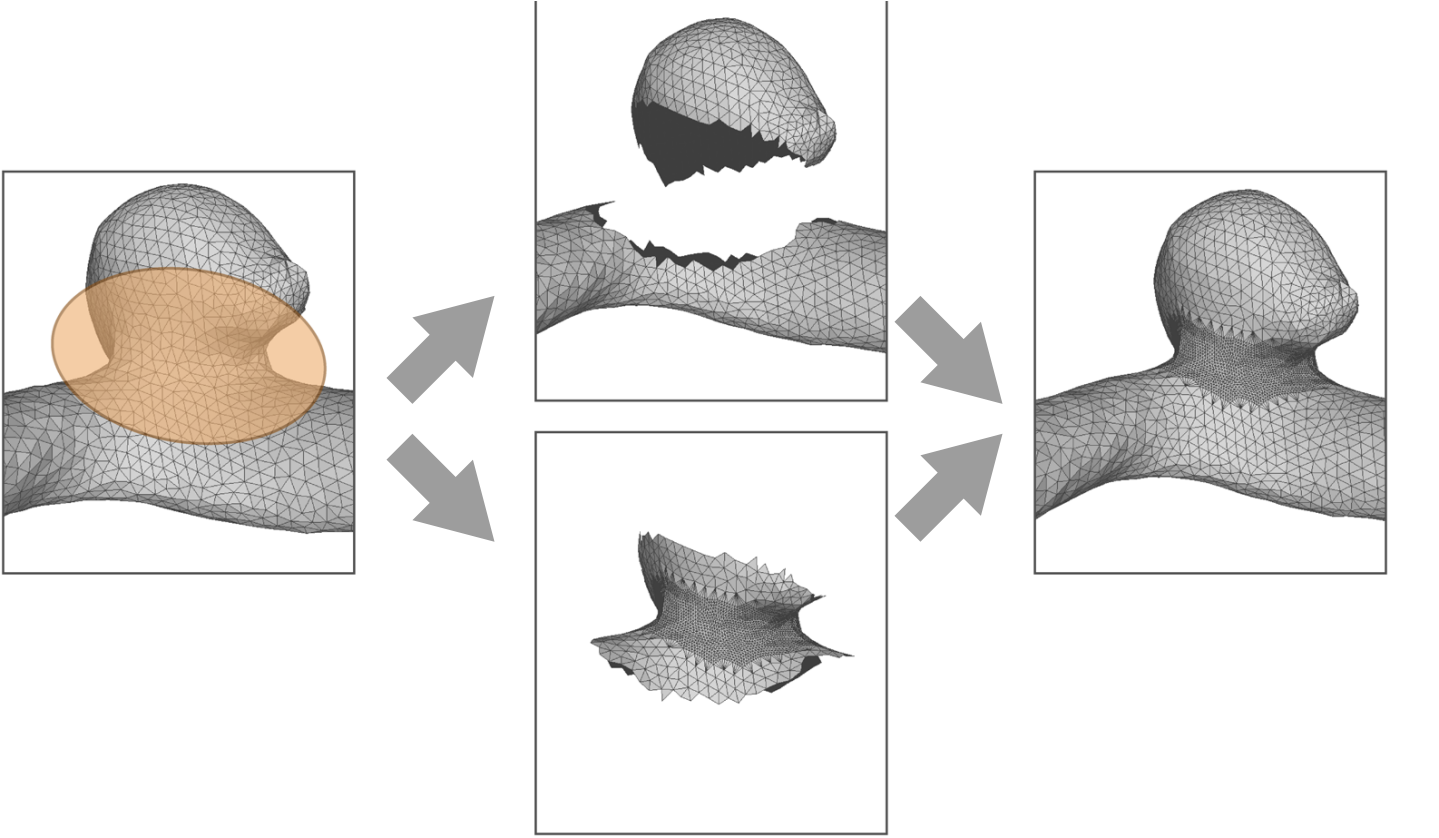
Illustration of the subdivision of triangles within the neck region: for each model, we cut the neck region and subdivided it such that the influence of the triangle size of the neck curve course is drastically reduced. Afterwards, the subdivided neck and the rest of the model were merged into a single model.

To reduce the influence of the segmentation masks on the neck curve path, we reduced the triangle size by subdivision of the neck for each model, this is illustrated in Fig. 3. The subdivision was restricted to the neck region only, to keep the performance of the web-based application as fast as possible.

#### Ostium Creation and Manipulation

After submitting the neck curve to the server, the associated ostium surface mesh was calculated automatically. Next, the manually defined border points were replaced by the neck curve points and simple Laplacian smoothing was applied to all vertices except the border points yielding a smooth and realistic initial ostium surface.

The second and optional task comprised of the adjustment of the previously calculated ostium surface. Here, the segmented vessel surfaces are illustrated with back faces only to reveal the ostium and allow a perception of its shape. The user was able to grab and drag any vertex on the ostium surface that was not part of its border (neck curve), recall Fig. 2.

#### Participants Registration

After submitting a neck curve and the corresponding ostium for each of the five IA cases, participants were requested to fill out a questionnaire (see supplementary material). Specifically, participants were asked to enter their name, e-mail address, occupation and affiliation. Furthermore, the individual experience related to IA treatment or research was queried. Thus, an examination of differences between user groups (e.g., physicians vs. engineers) becomes feasible.

For further details regarding the technical implementation of the VICTORIA web application the interested reader is referred to Behrendt *et al.*^2^

### Hemodynamic Simulations

To evaluate the effect of varying aneurysm sac-vessel-separations, image-based blood flow simulations were carried out using computational fluid dynamics (CFD). Specifically, three neck curves per case were considered to identify the minimum, median and maximum aneurysm surface area. Prior to each simulation, the IA model was spatially discretized using STAR-CCM+ 2020.01 (Siemens Product Lifecycle Management Software Inc., Plano, TX, USA) with a cell base size of $\varDelta x=0.08\,{\text{mm}}$ resulting in a total number of cells (polyhedral and prism layers) ranging from 1.2 to 2.6 million depending on the size of the considered vasculature.

Since no patient-specific boundary conditions were available, measured flow rates acquired using 7T phase-contrast MRI were scaled to the corresponding inlet cross sections.^5^ For each outlet, an advanced flow-splitting technique was applied.^11^ Blood was considered as a single-phase, incompressible ($\rho = 1055\,\frac{\text{kg}}{\text{m}^{3}}$) and Newtonian ($\eta = 4\,{\text{mPa s}}$) fluid and laminar flow conditions were assumed. To achieve a periodic solution, each time-dependent blood flow simulation comprises of three cardiac cycles (time step size $\varDelta t=0.001\,{\text {s}}$), while only the last one was included in the analysis.

### Analysis

The contributions submitted by each participant were qualitatively and quantitatively evaluated. First, the pairwise differences of two neck curves were extracted from user *i* and user *j*. Here, the squared distances were used, i.e.:

$$D^{\prime}_{i,j} = \sqrt{\sum ^n_{i=1}{{\text{min dist}}(p_{i},q_{j})^2}}$$

where ${\text{min dist}}(p_i,q_j)$ corresponds to the minimum distance for each point $p_i$ of the neck curve of user *i* comprising *n* points to all points $q_j$ of the neck curve of user *j* comprising *m* points. To account for variances between *n* and *m*, we extracted the pairwise difference $D_{i,j}$ as

$$D_{i,j} = \frac{1}{2} (D^{\prime}_{i,j} + D^{\prime}_{j,i}) = D_{j,i}$$

The pairwise differences were analyzed regarding all users as well as only for users with and without medical background, respectively. When adding all differences *D* of a single user to the remaining ones, it was possible to identify the user with smallest differences. Hence, the corresponding neck curves as *median* neck curve, i.e., the neck curve, which had the smallest cumulative distances to all other neck curves, could be defined.

Regarding the effect of variable neck curve definitions on hemodynamic predictions, the qualitative comparison focuses on time-averaged wall shear stress (*AWSS*). Additionally, quantitative analyses were carried out for all five cases with respect to neck inflow rate ($Q_{\text{in}}$), cycle-averaged wall shear stress ($\overline{AWSS}$) and mean and maximum oscillatory shear index ($\overline{OSI}$/${OSI}_{\text{max}}$), respectively. Further details regarding the parameter definitions can be found in Cebral *et al.*^10^

## Results

In total, 55 participants from 18 countries (Belarus, Bulgaria, Chile, France, Germany, Greece, Hong Kong, Hungary, India, Iran, Italy, Japan, Norway, Russia, Singapore, Sweden, Syria, U.S.A.) submitted their neck curve and ostium results. Among them, 20 were physicians with either neuroradiological or neurosurgical expertise. The remaining participants, associated to the non-clinicians' group, were mostly biomedical engineers with experience in aneurysmal research. Some persons reported knowledge in both fields and were assigned to one group according to their affiliation.

During participation, we also asked for additional information, regarding the years of experience, the number of aneurysms that had been evaluated so far (divided into categories $1{-}10$, $10{-}50$, $51{-}100$, $100{-}500$, $>500$), and the subjective rating of the importance of the ostium, rating from 1 (”Not important at all”) to 6 (”Very important, clearly affects the outcome”). The questionnaire is provided in the supplemental material. The physicians’ years of experience ranged from 1 to 29 years (average = 8.45 years) and the majority of them (11 out of 20) rated the ostium to be very important, which clearly affects the outcome. Most physicians stated that they already treated 10–50 aneurysms (6 out of 20). Twelve participants chose higher categories (5 out of 20 chose 51–100, 5 out of 20 chose 100–500 and 2 out of 20 chose > 500), and two participants stated that they only treated less than 10 aneurysms.

### Qualitative Comparison

Overall, the participants demonstrated a high similarity with respect to the individual neck curve selections and only a few outlying solutions were submitted. Hence, for the majority of clinically relevant aspects this would result in an appropriate aneurysm-vessel-separation.

**Figure 4:**
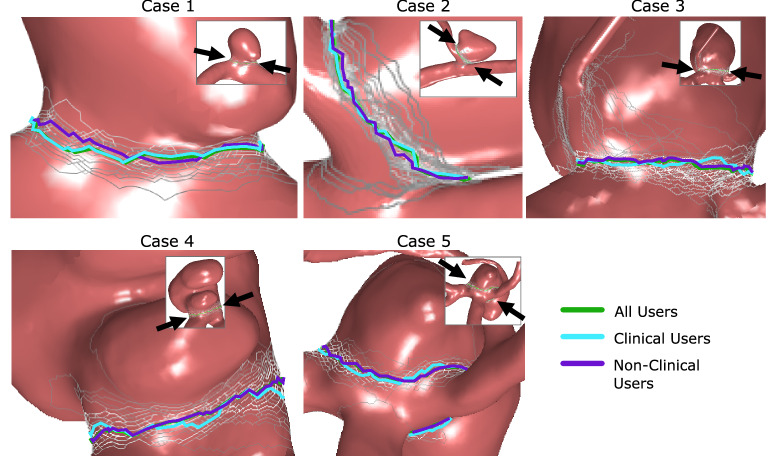
Illustration of the median neck curves for all five aneurysms of all users (green), the clinical users (cyan) and the non-clinical users (violet) showing a high agreement. The small inlays with arrows provide context information, which part of the aneurysm is shown. Furthermore, for each view, the most similar neck curve to the median neck curve is color-coded in white, the least similar neck curve is color-coded in gray and linear interpolation of colors-value is carried out in between based on all users.

Nevertheless, with increasing complexity of the case, the deviations among the different participants rise, see Fig. 4. The best agreement of neck curves was achieved for Case 1, i.e., the lateral IA with a relatively smooth transition from the parent vessel to the sac. Hence, participants (even with limited experience) managed to identify almost the same curve since it was a side wall aneurysm with good accessibility.

In the second case, the difficulty was increased by a small side branch adjacent to the aneurysm. This resulted in visual deviations for a few participants, especially when the neck was defined on the main vessel instead of the side branch. However, the overall range demonstrates that the defined neck curves were in a good agreement.

Larger differences were clearly visible for Case 3, which was a complex bifurcation aneurysm. Here, inconsistencies resulted from the existence of a small vessel close to the neck region. Specifically, some participants included the side branch, while it was excluded by others. Furthermore, outlying solutions occurred with clear distance to the neck regions.

The observations of Case 3 can be confirmed for Case 4 showing a relatively broad range of potential neck curves. Nevertheless, due to the presence of a lateral aneurysm, the number of outliers is limited and the variation of the neck curves rather relates to the distance to the parent vessel.

Finally, Case 5 (bifurcation aneurysm) experiences clear differences in the neck curve definitions as well. Especially in regions that are not in the vicinity of the adjacent vessels, larger deviations are present.

For the inclusion of side branches, we observed that in the majority of cases, the neck curve is defined at the narrowing between parent vessel and aneurysm. Very small arteries arising directly from the aneurysm (e.g. as present in Case 3) were mostly included. Larger arteries were mostly excluded (e.g. as it is shown for Case 2).

Regarding the direct comparison of the two sub-groups (clinicians vs. non-clinicians), no differences in the median neck curves were observed from a qualitative point of view. Instead, a good agreement among the groups can be noted, see Fig. 4.

**Figure 5:**
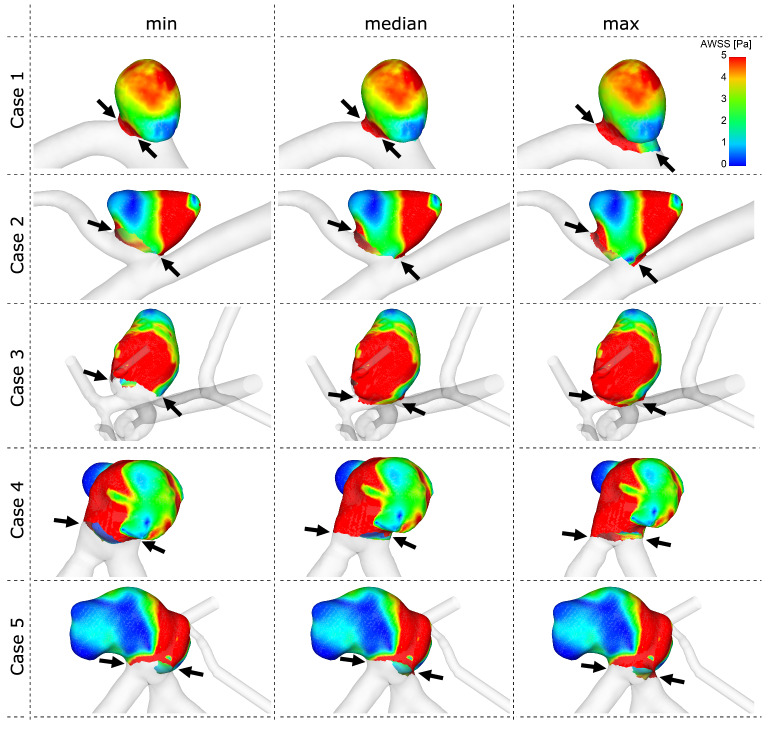
Qualitative comparison of the time-averaged wall shear stress (AWSS) prediction depending on the neck curve definition. Notice the differences occurring at the transition between each aneurysm and the corresponding parent vessel (marked by black arrows).

Since not only the actual differences between the participants solutions were of interest, the effect on the hemodynamic predictions was assessed. As demonstrated in Fig. 5 for the time-averaged wall shear stress, a careful selection of the neck curve is crucial for an accurate quantification. High shear stress values and gradients are noticeable at the aneurysm neck and depending on the participants choice, these patterns can be included in the quantitative analysis or not. This observation is specifically predominant for aneurysms with an adjacent side branch since it complicates an appropriate neck curve selection.

### Quantitative Comparison

**Figure 6:**
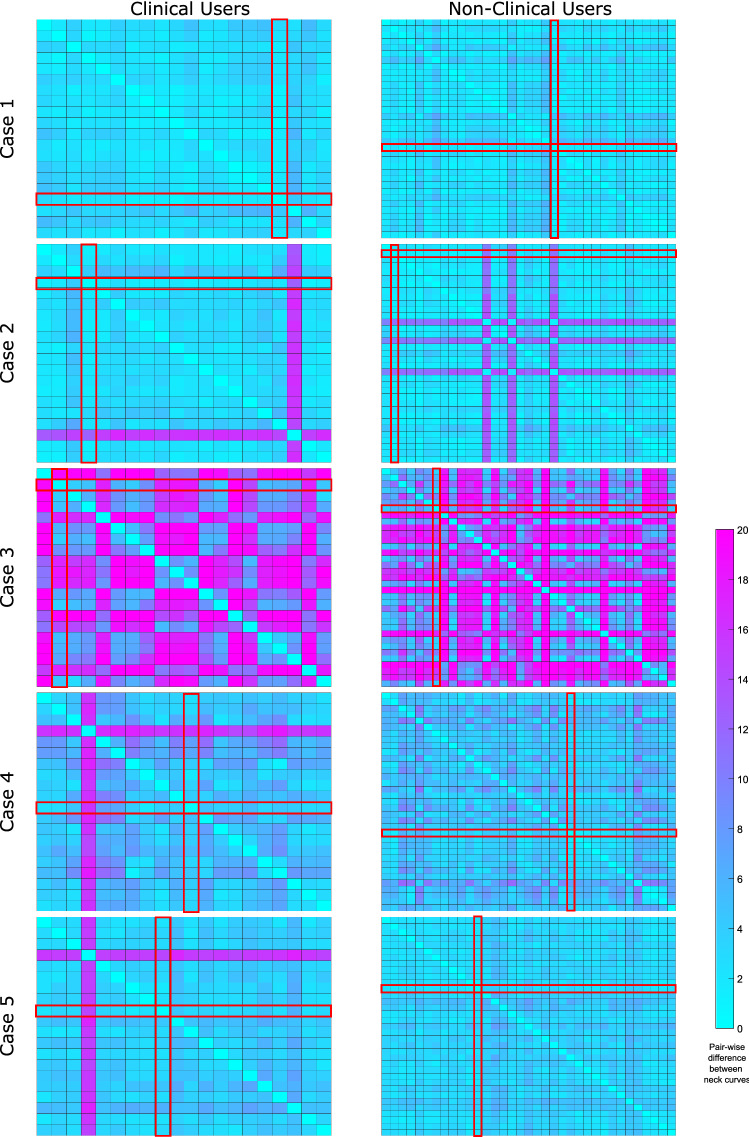
Heatmap-based illustration of the pairwise comparison of neck curves. Each matrix (i.e. the heatmap) color-codes the difference between the users. On the left column, only clinical users are listed, on the right, the non-clinical users are presented for each aneurysm case. The color-coding is kept constant for all cases to allow for a visual comparison. Each cell $c_{i,j}$ of a heatmap depicts the distance between the neck curve from user *i* and the neck curve from user *j*. The neck curves with smallest distances to all other neck curves are selected as median neck curves (highlighted in red). The overview shows strong agreement for aneurysm Case 1 and very poor agreement for Case 3, the most complex case. Also, there is one outlier for Case 2, Case 4 and Case 5 among the clinical users and three outliers for Case 2 among the non-clinical users. A detailed listing of all users for all cases including quantitative results is provided in the supplemental material.

The visual findings are confirmed when it comes to the quantitative analysis. Based on the pairwise differences (recall “Analysis” section), we extracted a matrix for each aneurysm case and each user group, i.e. for all users, for the clinical users and for the non-clinical users. The neck curve that has the smallest sum of distances compared to all other neck curves is identified as median neck curve. In Fig. 6, these matrices and the corresponding median neck curves are presented as heatmaps for the clinical and non-clinical users. The heatmaps for all user groups including the exact quantitative values are provided in the supplemental material. It can be noted that the qualitative observations are affirmed especially with respect to the type of aneurysm. While the best agreement is presented for a lateral IA with a well-defined neck region (Case 1), strongest variations occurred for the complex malformation located at a bifurcation (Case 3).

It can be observed that rather homogeneous distributions are present for Cases 1 and 3, while clear outlying solutions occur for the Cases 2, 4, and 5. Median neck curves for Cases 1 and 2 were defined by a medical expert, whereas for Cases 3, 4, and 5 biomedical engineers submitted the median solutions.

In addition to the distance quantification, variations with respect to relevant hemodynamic predictions were assessed (see Table 2). Specifically, the flow-related parameter neck inflow rate ($Q_{\text{in}}$) can vary up to one third depending on the ostium definition (e.g., Case 4), but is in a similar range for most neck curve selections. Hence, the calculation of this integral value is rather robust (e.g., Cases 2 and 5) with only minor differences of around 1–6%. Compared to the flow rate quantification, relevant shear-related parameters revealed stronger variations due to different neck curves. Here, the cycle-averaged wall shear stress of the minimum/maximum aneurysmal area was $11.4\pm 7.9\%$/$10.6\pm 4.9\%$ lower/higher compared to the median solution. The oscillatory shear index revealed a clear case-dependency since it is important where regions of increased values occur. While for most aneurysms (Cases 2–5) higher OSI is visible in the dome region, neck curve variability has only minor influence on mean or maximum values. However, if the largest values are present in the vicinity of the aneurysm neck (e.g., Case 1), non-neglectable differences in the risk assessment exist.

**Table 2:** Effects of the neck curve variability on the aneurysm surface $A_a$ and hemodynamic predictions using image-based blood flow simulations.

Case		$A_{a}$ [${\text{cm}}^{2}$]	$Q_{\text{in}}$ [mL/s]	$\overline{AWSS}$ [Pa]	$\overline{OSI} \cdot 10^{-3}$ [–]	$OSI_{\text{max}}$ [–]
1	min	0.274 (− 3.5%)	0.483 (− 0.9%)	10.856 (− 8.2%)	0.659 (− 1.1%)	0.069 (+ 0.9%)
	median	0.284	0.479	11.823	0.666	0.069
	max	0.312 (+ 10%)	0.589 (+ 23.1%)	13.573 (+ 14.8%)	0.884 (+ 32.7%)	0.131 (+ 90.4%)
2	min	0.480(− 5.4%)	0.426 (− 2%)	4.738 (− 3.3%)	1.890 (0%)	0.385 (+ 1.4%)
	median	0.508	0.435	4.900	1.890	0.380
	max	0.528(+ 4.0%)	0.426 (− 2%)	5.326 (+ 8.7%)	2.250 (+ 19%)	0.385 (+ 1.4%)
3	min	4.020 (− 13.2%)	6.064 (− 21.7%)	3.722 (− 23.4%)	81.150 (+ 9.7%)	0.490 (− 0.2%)
	median	4.632	7.749	4.862	73.970	0.491
	max	4.803(+ 3.7%)	6.222 (− 19.7%)	5.206 (+ 7.1%)	71.820 (− 2.9%)	0.491 (0%)
4	min	1.101 (− 4.7%)	1.034 (− 33%)	6.258 (− 17.1%)	5.700 (− 1.6%)	0.424 (0%)
	median	1.154	1.543	7.547	5.790	0.424
	max	1.213(+ 5.2%)	1.887 (+ 22.2%)	8.887 (+ 17.8%)	5.700 (− 1.6%)	0.424 (0%)
5	min	2.158 (− 2.3%)	1.737 (− 1%)	2.901 (− 3.7%)	20.200 (+ 2%)	0.473 (0%)
	median	2.209	1.754	3.014	19.800	0.473
	max	2.290 (+ 3.7%)	1.867 (+ 6.4%)	3.157 (+ 4.7%)	19.300 (− 2.5%)	0.473 (0%)

Minimum, median and maximum solutions are compared for each case focusing on flow- and shear-related parameters, respectively: $Q_{\text{in}}$, neck inflow rate; $\overline{AWSS}$, cycle-averaged wall shear stress; $\overline{OSI}$/${OSI}_{\text{max}}$, mean and maximum oscillatory shear index. The relative difference with respect to the median solution is given in brackets

## Discussion

Although the multidisciplinary research effort related to rupture risk assessment and treatment support of IAs drastically increased over the last years and specific knowledge about morphological and hemodynamic phenomena could be obtained, a successful translation of these findings into a clinical environment is still lacking.^13^,^34^,^38^ The underlying reasons are manifold: First, only commercially available software tools can be used. Second, the medical community is rather conservative and (potential) improvements need to be evaluated carefully. Third, the corresponding studies involve several interdisciplinary working steps (e.g., imaging,^17^ image segmentation,^31^ blood flow modeling^3^), which require multiple assumptions and therefore might be error-prone.^33^ Fourth, even if all or at least most of these steps are appropriately conducted, inaccuracies can even occur during the post-processing, i.e., the separation of the aneurysm sac from the parent vessel. This is in particular crucial, since many questions of interest are affected (e.g., quantification of the aneurysm neck or relevant flow- and shear-related parameters^19^,^27^).

This is also reflected by the analysis of the hemodynamic results within this study. Specifically, a considerable variability could occur simply due to a different processing of the simulation results. These observations are in line with previous findings such as segmentation-dependent variations in energy loss calculations or differences in the neck inflow rate between successfully and unsuccessfully treated aneurysm patients.[31, 39] However, for most contributions within VICTORIA almost similar hemodynamic results were obtained and largest deviations were associated with outlying and clinically insufficient contributions. Nevertheless, the parameter of interest should be critically observed (e.g., increased shear close to the neck region) when the separation of the aneurysm from its parent vessel is realized. This is of special importance for normalization purposes, e.g., when the time-averaged wall shear stress of the aneurysm sac is referred to the one of the corresponding parent vessel. Depending on the neck curve, high shear stress could either contribute to the numerator or denominator, respectively, and hence considerably influences the apparently objective parameter.

Thus, for specific parameter analysis, the location of the corresponding areas of interest should be taken into account, e.g., wall shear stress values close to the aneurysm neck versus maximum oscillatory shear indices rather occurring in the dome region. A consulting of several experts for neck curve definition might also be a necessary pre-processing step.

Based on the presented results of this study, a clear trend regarding the neck curve variability becomes visible. While for relative simple IAs, which are located at a lateral position of the parent vessel, a good agreement with respect to the sac-vessel-separation occurred, the differences increased with increasing complexity of the malformation. Specifically, bifurcation IAs that were associated with an increased risk of rupture,^22^ demonstrate rather inconsistent neck curve definitions (e.g., Case 3). Here, a clearly broader range of neck curve solutions exists leading to considerable differences regarding a potential aneurysm assessment.

Another important finding of this study is the inclusion or exclusion of very small arteries next to the aneurysm, as it is present for Cases 2 and 3. It can be observed that the majority of all users excluded these small arteries when defining the neck curve of the IA. Only 4 of 55 users in total (1 of 20 users with clinical background and 3 of 35 users without clinical background) included the small side branch for Case 2 and 15 of 55 users (7 of 20 with clinical background and 8 of 35 without clinical background) included it for Case 3. Noteworthy, the diameter of the branching artery is larger for Case 2 than for Case 3 w.r.t. the corresponding parent artery, which might correlate with the larger number of users including the small vessel in Case 3. In previous research projects, this issue was discussed only with few collaboration partners and a consensus regarding the *true* neck definition was not established. Therefore, based on the presented results, it is recommended to explicitly refrain from considering small arteries that are in the vicinity of an aneurysm.

Regarding the sub-analysis of participants with medical and non-medical background, the findings reveal no advantage for one of these groups. While clinicians created the neck curves with minimum overall distance to the other solutions for Cases 1 and 2, biomedical engineers succeeded for the remaining IAs.

Beside the presented findings, several limitations exist with respect to this study: First, although already 55 medical and non-medical participants contributed their solutions in the frame of this comparison, a higher number of participants would further strengthen its significance. Second, the datasets were already pre-segmented due to feasibility reasons. Third, the analysis included the comparison of the provided neck curves only and refrained from an inclusion of the ostia surface areas at this stage. However, the assessment of differences with respect to the ostium representation is ongoing work. In this regard, the metrics introduced by Cárdenes *et al.*^8^ could be integrated to strengthen the quantitative analysis. Fourth, the number of IAs considered in the frame of VICTORIA is limited. Here, a compromise between the coverage of different levels of complexity and the temporal effort for each (voluntary) participant had to be identified in advance. Fifth, only participants who were willing to invest time and who consequently cared about the ostium actually participated in the study, leading to a trend in the positive rating of the importance of the ostium. Sixth, several assumption are required for the hemodynamic simulations. This includes the choice of boundary conditions and material properties, which can have an impact on the presented deviations. Seventh, our study represents a research prototype where the focus relies upon availability, i.e. using a web-based technique rather than on its application in clinical practice.

## Conclusion

VICTORIAs findings reveal the complexity of aneurysm neck curve definition, especially for bifurcation aneurysms. After evaluation of the contribution from 55 participants, it appears to be mandatory to carefully separate the aneurysm sac from the parent vessel in future studies to avoid inaccurate parameter quantification. Furthermore, it is strongly recommended to refrain from considering small side branches occurring close to the IA when defining the neck curve such that only the aneurysm sac is separated from the corresponding parent artery. This ensures a precise assessment of morphologically and hemodynamically relevant parameters without the introduction of considerable errors in the final step of a multi-disciplinary workflow. In addition, simple planes are insufficient for complex aneurysm cases. Ongoing future work will quantify the influence of the VICTORIA’s median neck curves compared to planar ostium definition on parameter values. Finally, it should be noted that the presented architecture could be easily adapted to other medical image processing questions that require 3D models and user interaction.

## Supplementary Information

Below is the link to the electronic supplementary material.

Electronic supplementary material 1 (PDF 45 kb)
