# Supplementary material for: VICTORIA: VIrtual neck Curve and True Ostium Reconstruction of Intracranial Aneurysms
Source: Cardiovasc Eng Technol. 2021 Jun 7;12(4):454–65. doi: 10.1007/s13239-021-00535-w (PMC8354974; doi:10.1007/s13239-021-00535-w)
Supplement: Supplementary file 1 — Electronic supplementary material 1 (PDF 45 kb) [file 13239_2021_535_MOESM1_ESM.pdf]

Question	Possible answers
How many years have you been working in the context of intracranial aneurysms?	0-99
Please rate the importance of the conceptual understanding of the aneurysms ostium for your work.	0 (not at all) -<br>6 (very important,<br>clearly affects outcome)
Please estimate the total number of intracranial aneurysms you have treated/investigated by yourself.	<10<br>10 - 50<br>51 - 100<br>101 - 500<br>>500
What is your major treatment strategy for intracranial aneurysms? *(only for medical practitioners)*	Endovascular<br>Surgical
Please describe in one sentence (own words) the meaning of the term 'ostium'.	*Free text*

Table 1 Questionnaire shown to the participants after completing all tasks.
